# Supplementary material for: Identification and characterization of a new ensemble of cometary organic molecules
Source: Nat Commun. 2022 Jun 25;13:3639. doi: 10.1038/s41467-022-31346-9 (PMC9233696; doi:10.1038/s41467-022-31346-9)
Supplement: Supplementary file 1 — Supplementary Information [file 41467_2022_31346_MOESM1_ESM.pdf]

Identification and characterization of a new ensemble of cometary organic molecules

N. Hänni[1*], K. Altwegg[1], M. Combi[2], S. A. Fuselier[3,4], J. De Keyser[5], M. Rubin[1], and S. F. Wampfler[6]

[1] Physics Institute, Space Research & Planetary Sciences, University of Bern, Sidlerstrasse 5, 3012 Bern, Switzerland
[*]e-mail: nora.haenni@unibe.ch
[2] Department of Climate and Space Sciences and Engineering, University of Michigan, Ann Arbor, MI, USA
[3] Space Science Directorate, Southwest Research Institute, San Antonio, TX, USA
[4] Department of Physics and Astronomy, The University of Texas at San Antonio, San Antonio, TX, USA
[5] Royal Belgian Institute for Space Aeronomy, BIRA-IASB, Brussels, Belgium
[6] Center for Space and Habitability, University of Bern, Gesellschaftsstrasse 6, 3012 Bern, Switzerland

Supplementary material

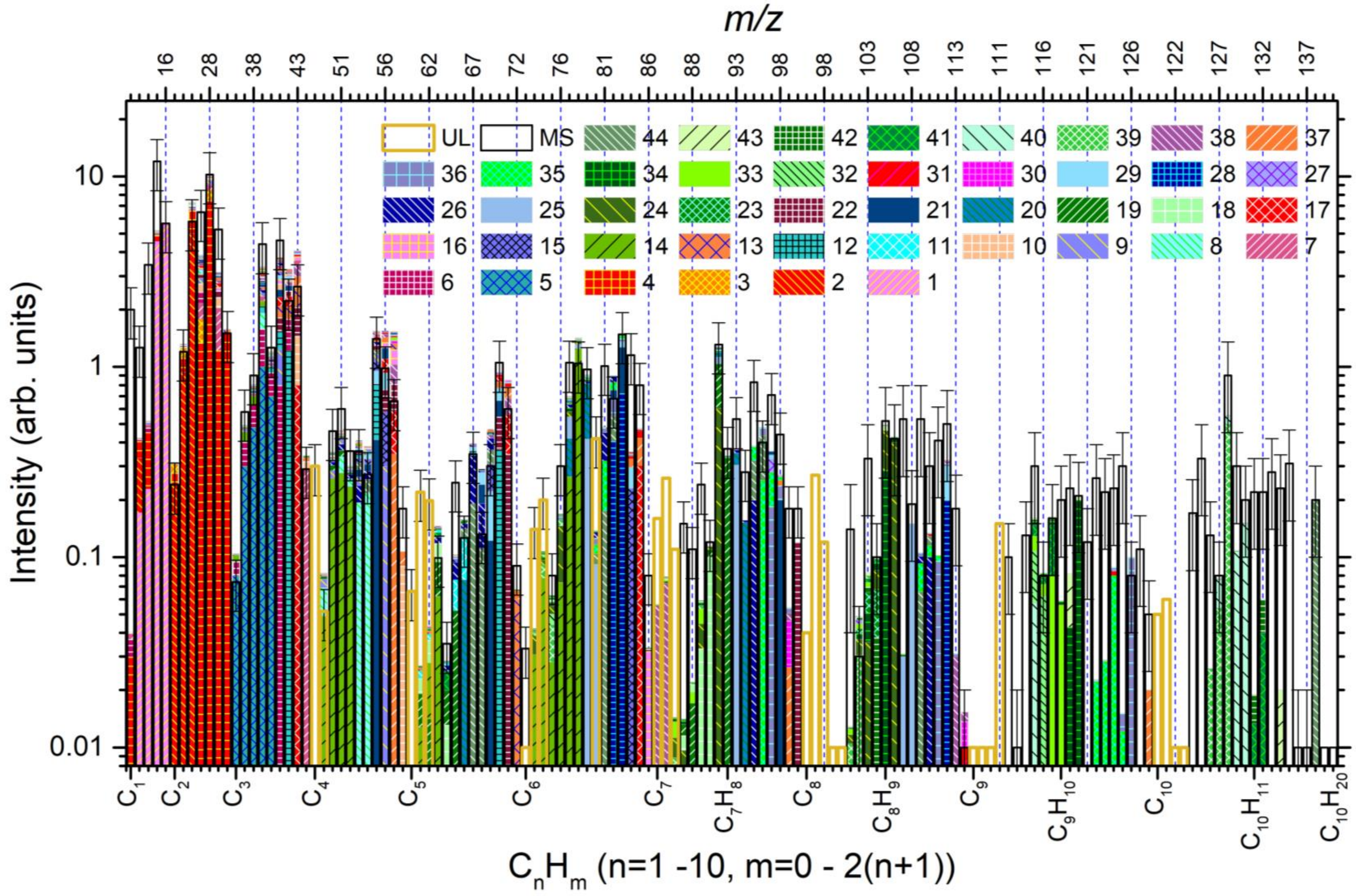

Supplementary Fig. 1: Deconvolved total intensity of pure hydrocarbon species detected by the Double Focusing Mass Spectrometer (DFMS) on 3 August 2015. Color-coded we show an Occam's razor conform deconvolution of the measured signals (MS; black bars) into individual fragmentation patterns of neutral pure hydrocarbons (numbering according to Table 1 of this article): chains (shades of red), cyclic species (shades of blue), and aromatic species (shades of green). Error bars indicate an estimated error of 30% on intensities. Upper limits (UL; yellow bars), due to interference of species with low H counts (small m) with mainly sulphur-bearing species, are shown in yellow.

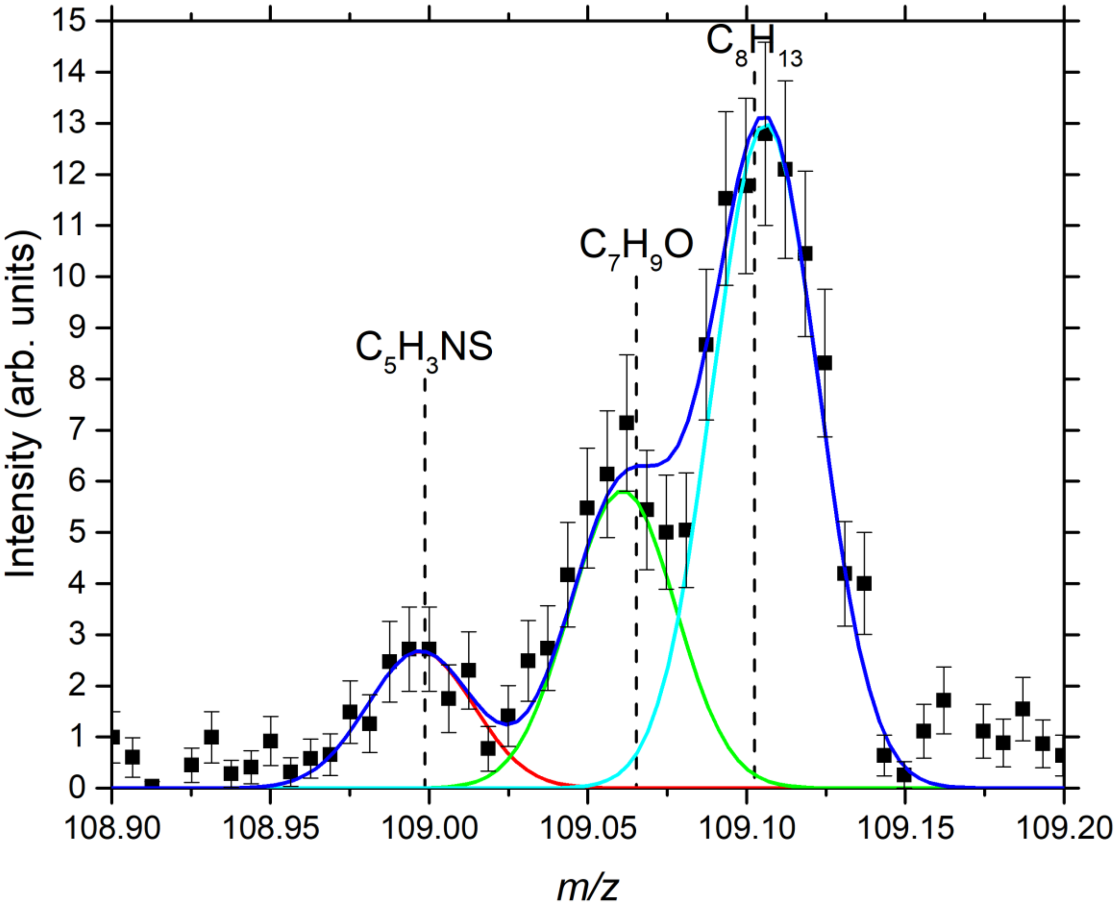

Supplementary Fig. 2: Mass spectrum collected with the Double Focusing Mass Spectrometer (DFMS) around integer *m/z* = 109. This example spectrum, recorded on 3 August 2015 (packet time: 08/03/2015 15:46:00.472), demonstrates the DFMS' capability to separate signals of pure hydrocarbon species from signals of heteroatom-bearing species. DFMS signals are generally best fit with a combination of two Gaussian functions, g_1 and g_2, whereby g_2 is roughly three times as broad but only 0.1 times as high as g_1. For weak signals, such as those in this plot, single Gaussians are used (here shown in red, green, and cyan) as the second Gaussian disappears in the background. The sum curve (blue) corresponds to the best fit to the spectrum. Error bars indicate 1σ statistical uncertainty.

Supplementary Table 1. List of molecules identified in data collected on 3 August 2015 with the Double Focusing Mass Spectrometer (DFMS). Molecules were identified either via full deconvolution of the pure hydrocarbon subset of data (pure hydrocarbon species) or via characteristic finger print features (heteroatom-bearing species). The reasoning of the identification is justified and judged regarding its level of confidence. References for the employed fragmentation patterns are included. For compounds that have not been calibrated on the DFMS laboratory twin instrument at the University of Bern, we refer to fragmentation patterns available from the National Institute of Standards and Technology (NIST).

Parent formula (C_nH_m)	Molecule	Reference data	Reason for inclusion (numbers indicate *m/z* values and M stands for molecular ion)	Likeliness rating
CH_4	Methane	calibrated	M at 16	certain
C_2H_2	Acetylene	calibrated	M at 22	certain
C_2H_4	Ethylene	NIST	M at 24	certain
C_2H_6	Ethane	calibrated	M at 30	certain
C_3H_4	Cyclopropene	NIST	ratio 39/40	better fit than for propyne
C_3H_6	Propene	NIST	ratio 41/42	certain
C_3H_8	Propane	calibrated	M at 44	certain
C_4H_6	Cyclobutene	NIST	M at 54, ratio 53/54	1,3-Butadiene possible, slightly worse fit
C_4H_8	Cyclobutane	NIST	M at 56	Butene equally probable
C_4H_{10}	Butane	calibrated	M at 58	certain
C_5H_6	Cyclopentadiene	NIST	M at 66	very likely, better fit than for other isomers
C_5H_{10}	Cyclopentane	NIST	M at 70, major fragment at 55	very likely, better fit than for other isomers
C_5H_{12}	Pentane	calibrated	M at 72	certain
C_6H_6	Benzene	calibrated	M at 78, major fragment at 77	certain
C_6H_{12}	Cyclohexane	NIST	M at 84	very likely, better fit than for other isomers
C_6H_{14}	Hexane	calibrated	M at 86	certain
C_6H_{14}	Isohexane	NIST	major fragment at 71	very likely, no good alternative
C_7H_6	Bicyclo[4.1.0]hepta-1,3,5-triene	NIST	M at 90, major fragment at 89	very likely, no good alternative
C_7H_8	Toluene	NIST	M at 92, major fragment at 91	certain
C_7H_{10}	1,3-Cycloheptadiene	NIST	M at 94, major fragment at 79	very likely, no good alternative
C_7H_{14}	Methylcyclohexane	NIST	M at 98, major fragment at 83	very likely, no good alternative
C_7H_{16}	Heptane	calibrated	M at 100	certain
C_8H_8	Styrene	NIST	M at 104	other isomers possible
C_8H_{10}	p-Xylene	NIST	M at 106, major fragment at 91	very likely, no good alternative
C_8H_{12}	3-Ethenylcyclohexene	NIST	M at 108, major fragment at 93	very likely, no good alternative

C_8H_{14}	1,2-Dimethylcyclohexene	NIST	M at 110, major fragments at 95, 81, 67	very likely, other isomers have low 110/81 and 110/67
C_8H_{16}	1,1-Dimethylcyclohexane	NIST	major fragment at 97	likely, other isomers with methyl groups possible
C_8H_{16}	Ethylcyclohexane	NIST	strong M at 112, major fragment at 83	likely, no good alternative for this combination
C_8H_{16}	Cyclooctane	NIST	M at 112	likely because it can fill up 112
C_8H_{18}	2,5-Dimethylhexane	NIST	M at 114, major fragment at 112	likely
C_8H_{18}	Octane	calibrated	major fragment at 71	likely
C_9H_8	Indene	NIST	M at 116, major fragment at 115	very likely
C_9H_{10}	Indane	NIST	M at 118, major fragment at 117	very likely
C_9H_{12}	Mesitylene	NIST	M at 120, major fragment at 105	very likely
C_9H_{16}	Octahydro-1H-indene	NIST	M at m/z 124 and major fragments at 112, 123 and 96,97	likely
C_9H_{18}	1,2,3-Trimethylcyclohexane	NIST	M at m/z 126 and major fragments at111, 97 and 83	likely
C_9H_{20}	Nonane	NIST	M at 128, fragment at 99, 85, 71	certain
C_9H_{20}	2-Methyloctane	NIST	fragment at 113	likely
$C_{10}H_8$	Naphthalene	calibrated	strong M at 128	certain
$C_{10}H_{10}$	1,2-Dihydronaphthalene	NIST	M at 130, major fragments at 129, 115	indistinguishable from 1-methyl-1H-indene
$C_{10}H_{12}$	2,3-Dihydro-2-methyl-1H-indene	NIST	M at 132, major fragment at 117	probable
$C_{10}H_{12}$	1,2,3,4-Tetrahydronaphthalene	NIST	M at 132, major fragment at 104	probable
$C_{10}H_{14}$	1,4-Diethylbenzene	NIST	M at 134, major fragment at 119	likely
$C_{10}H_{18}$	Decahydronaphthalene	NIST	M at 138, major fragments at 109, 96, 95, 82, 81	very likely

**Parent formula (C_nH_mO)**	**Molecule**	**Reference data**	**Reason for inclusion (numbers indicate *m/z* values and M stands for molecular ion)**	**Likeliness rating**
C_4H_4O	Furane	NIST	M at 68, no signal at 67 (M-H)	likely due to presence of the hydrogenated derivatives
C_4H_6O	Dihydrofuran	NIST	M at 70, major fragment at 69 (M-H)	very likely, various positions of the double-bond
C_4H_8O	Tetrahydrofurane	NIST	M at 72, major fragment at 71 (M-H)	very likely
C_7H_6O	Benzaldehyde	NIST	M at 106, major fragments at 105 (M-H), 77 (C_6H_5)	very likely
C_7H_8O	Benzylalcohol	NIST	M at 108, major fragments at 107 (M-H), 79 (C_6H_7), 77 (C_6H_5)	very likely

**Parent formula ($C_nH_mO_2$)**	**Molecule**		**Reason for inclusion (numbers indicate *m/z* values and M stands for molecular ion)**	**Likeliness rating**
$C_3H_6O_2$	Propanoic acid	NIST	M at 74, major fragments at 73 (M-H), 45 (COOH)	very likely
$C_7H_6O_2$	Benzoic acid	NIST	M at 122, major fragments at 105 (M-OH), 77 (C_6H_5)	certain

**Parent formula (C_nH_mN)**	**Molecule**		**Reason for inclusion (numbers indicate *m/z* values and M stands for molecular ion)**	**Likeliness rating**
C_4H_9N	Pyrrolidine	NIST	M at 71, major fragments at 70 (M-H), 43 (likely C_3H_7)	likely
C_6H_9N	Dimethylpyrrole	NIST	M at 95, major fragments at 94 (M-H), 80 (M-CH_3)	very likely, various positions of the methly-group
C_7H_5N	Benzonitrile	NIST	M at 103, no signal at 102 (M-H), major fragment at 76 (C_6H_4)	likely
C_7H_9N	Benzylamine	NIST	M at 107, major fragments at 106 (M-H), 91 (M-NH_2), 79 (C_6H_7), 77 (C_6H_5)	certain

**Parent formula (C_nH_mS)**	**Molecule**		**Reason for inclusion (numbers indicate *m/z* values and M stands for molecular ion)**	**Likeliness rating**
C_4H_6S	Dihydrothiophene	NIST	M at 86, major fragment at 85 (M-H)	various positions of the double-bond, indistinguishable from divinylsulfide

**Parent formula ($C_nH_mS_2$)**	**Molecule**		**Reason for inclusion (numbers indicate *m/z* values and M stands for molecular ion)**	**Likeliness rating**
CH_4S_2	Methylhydrogendisulfide	NIST	M at 80, major fragments at 79 (M-H), 78 (M-2H), 46 (CH_2S)	very likely, for methanedithiol no MS data is available
